# Anodal tDCS improves the effect of neuromuscular training on the feedforward activity of lower extremity muscles in female taekwondo athletes with dynamic knee valgus

**DOI:** 10.1038/s41598-024-70328-3

**Published:** 2024-08-28

**Authors:** Mozhdeh Sadat Moshashaei, Farzaneh Gandomi, Ehsan Amiri, Nicola Maffulli

**Affiliations:** 1 Department of Sports Injuries and Corrective Exercises, Faculty of Sport Sciences, Razi University, Kermanshah, Iran; 2 Exercise Metabolism and Performance Lab (EMPL), Department of Exercise Physiology, Faculty of Sport Sciences, Razi University, Kermanshah, Iran; 3 Department of Trauma and Orthopaedic Surgery, Faculty of Medicine and Psychology, University La Sapienza, 00185 Roma, Italy; 5 School of Pharmacy and Bioengineering, Keele University School of Medicine, Stoke on Trent ST4 7QB, Staffordshire, UK; 4 Centre for Sports and Exercise Medicine, Barts and the London School of Medicine and Dentistry, Mile End Hospital, Queen Mary University of London, London E1 4DG, UK

**Keywords:** Neuroscience, Physiology, Health care, Nanoscience and technology

## Abstract

Transcranial direct current stimulation (tDCS) can increase cortical excitability of a targeted brain area. This study aimed to investigate the effect of adding anodal-tDCS (a-tDCS) to neuromuscular training (NMT) on the dynamic knee valgus (DKV) and feedforward activity (FFA) of knee muscles. Thirty-four Taekwondo athletes with DKV, were randomly assigned to either NMT + a-tDCS (N = 17) or NMT + sham tDCS (N = 17). DKV and the knee muscles' FFA at the moment of single and double-leg landing and lateral hopping tasks were evaluated before and after the interventions. DKV and FFA of the knee muscles was improved in all tasks (*P* < 0.05), however, between-group differences were not significant (*P* > 0.05). The FFA of the semitendinosus, vastus medialis, gluteus medius, and gastrocnemius muscles in the single-leg landing (*P* < 0.05), the gluteus medius, gluteus maximus, semitendinosus, biceps femoris, and gastrocnemius muscles in the double-leg landing (*P* < 0.05), and the gluteus medius, gluteus maximus, and gastrocnemius muscles in the lateral hopping (*P* < 0.05) tasks were significantly different between the groups. A-tDCS achieved significantly larger improvements in the feedforward activity of lower extremity muscles compared with sham-tDCS. However, between-group comparisons did not show a significant difference in DKV.

## Introduction

Anterior cruciate ligament (ACL) tears are one of the most common knee injuries that usually occur during tasks such as cutting and landing^1^. It has been reported that athletes participating in contact sports are at higher risk of ACL injuries^2^. For instance, in Taekwondo which is considered one of the most popular sports in many countries, ACL injuries have shown to be the most common injuries among these athletes^3–5^. Because of its nature, Taekwondo involves different kinds of jumping and kicking performance and interestingly, jumping kicks are reported to have a high risk of injury, particularly to the ACL^5,6^. When performing jumping kicks, one needs to make one leap, turn, rotate in the air, and land after kicking multiple targets at higher positions^7^, which increases the risk of non-contact ACL injuries^8^. In this regard, it has been reported that three out of five Taekwondo athletes have experienced knee joint ACL injuries at the moment of landing^9^.

Hewett et al. introduced 4 neuromuscular defects including trunk dominance, ligament dominance, quadriceps dominance, and foot dominance as underlying mechanisms of the ACL injury. Dynamic knee valgus (DKV) defect is ligament dominance, which is one of the most common neuromuscular defects that occurs when the necessary dynamic stability of the joint is not provided by neuromuscular control strategies^10^. Additionally, it is one of the abnormal movement patterns which places significant tensile on the ACL, and is widely accepted as one of the most important causes of the non-contact ACL injuries^11^, especially in the population of female athletes^2^. Females typically do not generate as much force as male counterparts in the hip abductors, thus potentially subjecting them to dynamic knee valgus moments^12^.

Neuromuscular control plays a vital role in supporting and stabilizing the knee joint by using the sensory inputs of mechanical receptors and regulating appropriate muscle responses^13^. It is also effective for lower limb biomechanics during motion tasks, especially in sensitive situations such as landing and cutting movements in which the respective joints are more loaded. During unpredictable movements, for example, hip muscles weakness can change landing kinematics and kinetics^14,15^. Changed or reduced neuromuscular control during the execution of sports tasks including excessive lower limb joint motions and loads may increase the risk of ACL injury in female athletes^16^. Knee stability is a complex interaction between static and dynamic restrictions in which neuromuscular control depends on an interaction of the visual, vestibular, and somatosensory systems^16^. Keeping dynamic knee joint stability during sports maneuvers requires a combination of pre-programmed muscle activity (feed-forward) and reflex-mediated muscle activity (feedback)^17^. Feedback process includes a class of short and long latency reactions driven by sensory data from numerous sources whereas feedforward neuromuscular control can be characterized as an individual’s expectation and/or planning for a particular action to successfully perform a task^17^. Feedforward mechanism is activated before foot contact after jumping (e.g., prelanding), and lower limb muscles are preactivated, presumably to prevent possible injuries. Accordingly, feedforward neuromuscular control deficiency such as inadequate muscle activation and/or joint kinematics before contact time could not limit the impact force during landing^18,19^. The time of ligament injuries during fast movements in sports is much shorter than the time that the feedback control system can deal with and counteract the incoming injuring forces so that the damage occurs before the muscles are activated in the form of feedback mechanisms^20^.

Neuromuscular training (NMT), has demonstrated statistically significant preventive role in ACL injuries^21–23^. Neuromuscular training is a comprehensive approach to strength and fitness training, integrating both sport-specific and foundational movements. This method incorporates a variety of exercises such as resistance training, balance, core strengthening, dynamic stability drills, agility exercises, and plyometrics^24^. It is unclear to what extent the various components of these programs contribute to reducing the risk of injury, however, neuromuscular exercises aimed at reducing DKV and improving single-leg stability and balance seem to play an essential role in this regard^25–27^. Neuromuscular exercises significantly reduce landing forces, knee valgus-varus torque and improve DKV, single-leg stability, and landing balance^27^. Nevertheless, the prevalence of ACL injuries is still high and this accentuates the importance of developing new strategies to tackle this situation.

Transcranial direct current stimulation (tDCS) has recently attracted attention in sports injury prevention and rehabilitation. This technique known as the most common type of non-invasive brain stimulation, a small electric current (0.5–2 milliamps), passing through the scalp by two electrodes (anode–cathode), changes the excitability of target areas in the brain^28^. It can expedite or suppress the excitability to find out neuro-physiological function in each area of the brain, and is being used in many different areas for recovery of performance^29^. The primary motor cortex (M1) is the brain region that has attracted the most attention given its importance in sports performance. The M1 area is involved in the development of motor programs and activation of the spinal motor neurons^30^. Anodal tDCS improves the performance of complex tasks such as skilled visuomotor tracking, muscle strength, and walking in the lower limb^31^. It has been shown that applying 15 min of anodal tDCS over the M1 areas representing the lower limb during a visuomotor ankle task improved the ankle choice reaction time^31^. In another study, Yang et al. investigated the effect of tDCS on the lower limb muscles’ activation and balance of soccer players. They reported a positive effect of tDCS on muscle activation and attributed it to tDCS alternating the activity of brain neurons in a way that is necessary for the recovery of motor function, promoting synaptic plasticity, and providing unspecific input for the brain motor cortex system^29^.

Taken together, the underlying mechanisms of DKV-induced ACL injuries such as unfavorably changed or reduced neuromuscular control, and tDCS-related mechanisms of improved neuromuscular system raise an interesting question as to whether the stimulation of the brain areas involved in neuromuscular control system could favorably improve neuromuscular control and counteract the detrimental effects of DKV. Moreover, while most of the tDCS-related studies have been conducted on male participants, recent findings have shown that there are sex differences in the brain’s responsiveness to tDCS^32^, indicating that caution must be exercised when generalizing the previous findings of male participants to females. Finally, there is a dearth of information in previous studies regarding the use of athletes performing specific sports (such as Taekwondo) in scientific studies. Hence, to bridge these gaps, we evaluated the effectiveness of 12 sessions of NMT in combination with anodal-tDCS (a-tDCS) on the feedforward activity of the lower extremity muscles among professional female Taekwondo players with DKV. To the best of our knowledge, this is the first study to examine the effects of tDCS combined with neuromuscular training in improving the feedforward activity of the lower extremity muscles of the knee in this target population. We hypothesized that adding a-tDCS to 12 sessions of NMT would increase the feedforward activity of the knee-stabilizing muscles in the female Taekwondo athletes with DKV.

## Results

A total of 34 female Taekwondo athletes were randomly assigned to either the experimental or control groups (17 subjects in each group). All the subjects received the planned research intervention for three sessions per week for four weeks. No subject dropped out of the study during the intervention period and in the post-intervention tests. The Shapiro–Wilk and Leven’s test results confirmed the assumptions of the parametric statistics (*P* > 0.05). The demographic characteristics and DKV of the subjects of the two groups were examined in the pre-test using the independent sample t-test, and no significant difference was observed between the groups (*P* > 0.05) (Table 1).

**Table 1:** Demographic and baseline characteristics (n = 34).

	Groups	Age (yrs.)	Weight (Kg)	Height (m)	BMI (kg/m^2^)	Training (h/w)	Training history (yrs.)	DKV (degree)
Mean ± SD	a-tDCS (n = 17)	21.52 ± 5.78	60.12 ± 8.89	1.64 ± 0.05	22.23 ± 2.62	5.30 ± 0.83	5.68 ± 1.90	15.05 ± 1.81
*P* value	s-tDCS (n = 17)	22.00 ± 4.88	58.17 ± 9.58	1.62 ± 0.05	22.12 ± 3.62	5.40 ± 0.83	5.41 ± 1.37	15.82 ± 2.98
		0.79	0.54	0.31	0.92	0.73	0.68	0.37

SD: standard deviation; a-tDCS: Anodal tDCS; s-tDCS: Sham tDCS. h/w: hours in a week; DVA: dynamic knee valgus.

### Dynamic knee *valgus*

Both the a-tDCS stimulation and the sham-tDCS (s-tDCS) groups after 12 intervention sessions significantly reduced the dynamic valgus angle in both single-leg landing and double-leg landing tasks (*P* < 0.05). The percentage change over time in the two study groups showed that the anodal stimulation group had a more favorable effect. However, the results of inter-group comparisons showed that the two groups of real and sham stimulation had no statistically significant difference in both single-leg landing and double-leg landing skills (*P* > 0.05) (Tables 2 and 3).

**Table 2:** Within–between groups comparisons of feedforward muscle activity for the single-leg landing task (a-tDCS = s-tDCS, n = 17).

Muscles	Groups	Baseline	Four-week	CP	CI 95%	Within group comparison	Between groups comparison	
		Mean ± SD	Mean ± SD			*P* value	*P* value	ɳ^2^_p_
DKV (^ô^)	a-tDCS	15.23 ± 2.65	12.41 ± 2.80	− 20.65	(2.43, 4.03)	0.0001**	0.18	0.55
	s-tDCS	15.64 ± 2.59	12.47 ± 2.34	–18.12	(1.81, 3.71)	0.0001**		
VL	a-tDCS	95.82 ± 36.29	141.70 ± 48.74	47.81	(− 61.51, − 30.59)	0.0001**	0.33	0.03
	s-tDCS	117.02 ± 33.53	129.47 ± 29.99	10.63	(− 16.28, − 8.59)	0.0001**		
VM	a-tDCS	89.89 ± 37.43	122.18 ± 41.63	35.92	(− 45.20, − 19.38)	0.0001**	0.18	0.057
	s-tDCS	106.97 ± 39.36	118.06 ± 43.23	10.36	(− 17.28, − 4.91)	0.002**		
Gmax	a-tDCS	60.96 ± 23.61	83.34 ± 27.04	36.71	(− 29.98, − 14.78)	0.0001**	0.014*	0.18
	s-tDCS	70.01 ± 20.13	34.80 ± 12.98	− 50.29	(− 15.20, − 6.01)	0.0001**		
Gmed	a-tDCS	63.32 ± 20.06	80.22 ± 21.47	26.68	(− 22.34, − 11.45)	0.0001**	0.04*	0.12
	s-tDCS	50.50 ± 15.35	62.02 ± 15.21	22.81	(− 17.89 to − 5.15)	0.001**		
ST	a- tDCS	41.97 ± 18.51	73.39 ± 32.21	74.86	(− 41.64, − 21.18)	0.0001**	0.01**	0.17
	s- tDCS	40.32 ± 11.23	51.21 ± 13.94	27	(− 16.67, − 5.09)	0.001**		
BF	a- tDCS	41.52 ± 21.71	62.86 ± 24.68	51.39	(− 28.20, − 14.45)	0.0001**	0.009**	0.2
	s- tDCS	59.75 ± 33.29	70.34 ± 29.40	17.72	(− 16.50, − 4.68)	0.002**		
Gas	a- tDCS	58.87 ± 24.30	93.01 ± 26.49	57.99	(− 46.10, − 22.16)	0.0001**	0.0001**	0.41
	s- tDCS	66.90 ± 30.41	86.63 ± 39.10	29.49	(− 28.97, − 10.48)	0.0001**		

All the muscles show the value of %MVIC; VL: Vastus lateralis; VM: Vastus Medialis; Gmax: Gluteus Maximus; Gmed: Gluteus Medius; ST: Semitendinosus; BF: Biceps Femoris; Gas: Gastrocnemius; CI: Confidence Interval; SD: Standard deviation; DKV: dynamic knee valgus; CP: Change percentage; ** = *P* < 0.01.

**Table 3:** Within–between groups comparisons of feedforward activity of muscles for the double-leg jump-landing task (a-tDCS = s-tDCS, n = 17).

Muscles	Groups	Baseline	Four-week	CP	%95 CI	Within group comparison	Between-	group comparison
		Mean ± SD	Mean ± SD			*P* value	*P* value	ɳ^2^_p_
DKV(^ô^)	a-tDCS	15.05 ± 1.18	11.00 ± 2.89	− 26.91	(2.94, 5.17)	0.0001**	0.06	0.1
	s-tDCS	15.82 ± 2.98	12.70 ± 3.29	− 19.72	(2.02, 4.20)	0.0001**		
VL	a-tDCS	117.44 ± 40.81	166.31 ± 50.90	41.61	(− 60.75, − 36.98)	0.0001**	0.06	0.1
	s-tDCS	109.34 ± 47.87	134.20 ± 56.14	22.73	(− 39.31, − 10.39)	0.002**		
VM	a-tDCS	103.50 ± 48.55	152.72 ± 54.98	47.55	(− 63.42, − 35.02)	0.0001**	0.01*	0.18
	s-tDCS	114.17 ± 47.04	126.88 ± 48.76	11.13	(− 19.48, − 5.94)	0.001**		
Gmax	a-tDCS	46.63 ± 24.16	76.63 ± 36.45	64.33	(− 44.89, − 15.09)	0.001**	0.11	0.07
	s-tDCS	60.74 ± 29.95	73.85 ± 27.83	21.58	(− 18.71, − 6.97)	0.0001**		
Gmed	a-tDCS	35.65 ± 19.92	71.30 ± 31.78	100	(− 45.26, − 26.04)	0.0001**	0.03**	0.18
	s-tDCS	31.72 ± 15.65	43.55 ± 18.25	37.2	(− 18.48, − 5.16)	0.002**		
ST	a-tDCS	30.89 ± 13.12	65.02 ± 34.81	110.48	(− 49.42, − 18.84)	0.0001**	0.04*	0.12
	s-tDCS	35.17 ± 16.80	50.87 ± 20.98	44.64	(− 23.52, − 7.87)	0.001**		
BF	a-tDCS	35.09 ± 19.83	66.12 ± 26.88	88.42	(− 41.45, − 25.60)	0.0001**	0.35	0.02
	s-tDCS	46.12 ± 26.78	53.78 ± 24.68	16.6	(− 26.07, − 5.75)	0.002**		
Gas	a-tDCS	40.42 ± 14.59	87.89 ± 34.65	117.44	(− 66.91, − 28.01)	0.0001**	0.0001**	0.36
	s-tDCS	48.97 ± 37.22	59.79 ± 36.83	22.09	(− 15.37, − 6.26)	0.0001**		

All the muscles show the value of %MVIC; VL: Vastus lateralis; VM: Vastus Medialis; Gmax: Gluteus Maximus; Gmed: Gluteus Medius; ST: Semitendinosus; BF: Biceps Femoris; Gas: Gastrocnemius; CI: Confidence Interval; SD: Standard deviation; CP: Change percentage; ** = *P* < 0.01.

### Feedforward knee muscles activity: single-leg landing task

**Figure 1:**
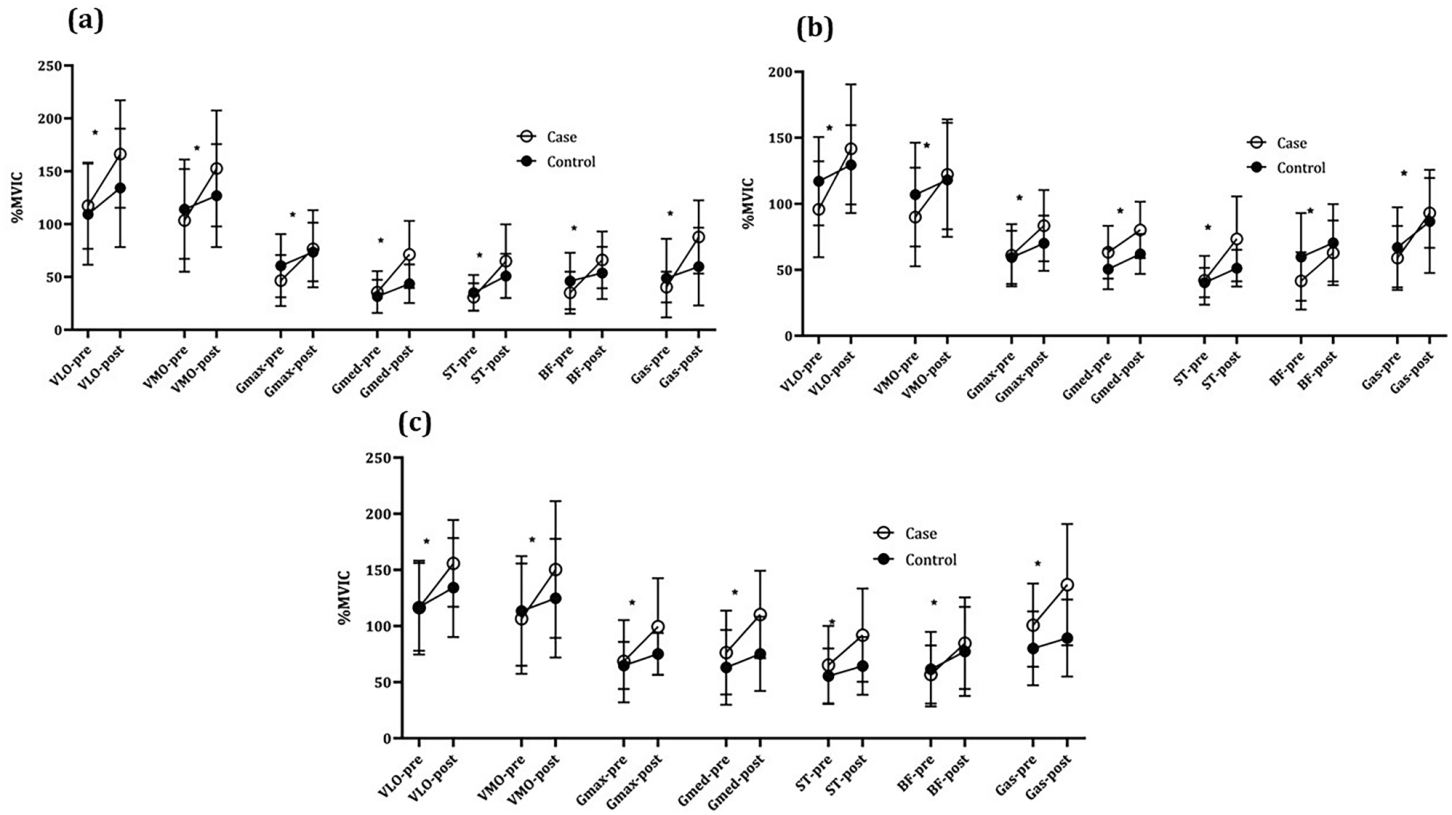
Within-groups muscles feedforward activity comparisons: (**a)** Double-leg landing task; (**b)** single-leg landing task; **c**. lateral hopping task (^*^ = *P* < 0.01).

**Figure 2:**
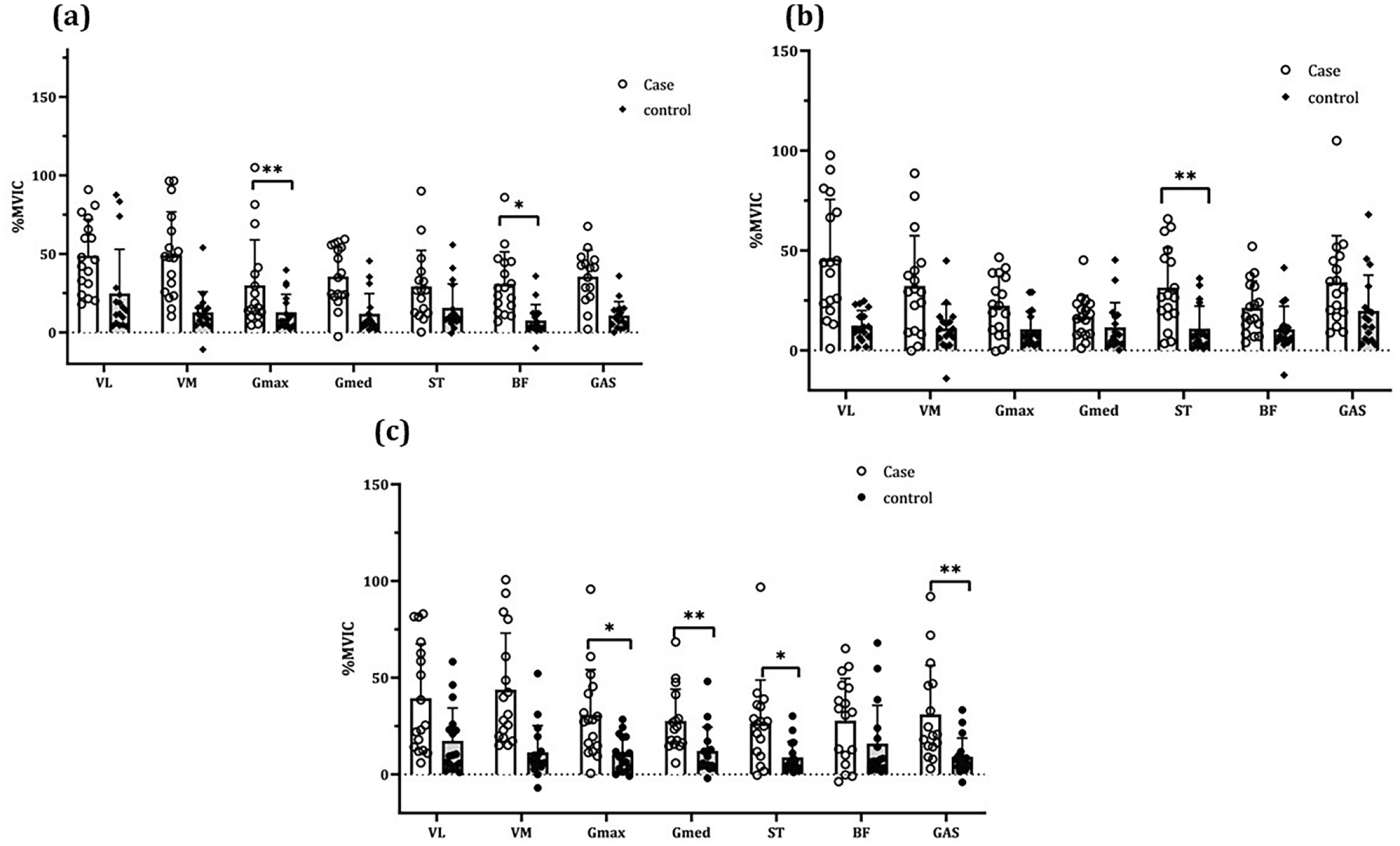
Between-groups muscles feedforward activity comparisons: (**a)** Double-leg landing task; (**b)** single-leg landing task; (**c)** lateral hopping task (^**^ = *P* < 0.01, ^*^ = *P* < 0.05).

The intra-group comparisons (Table 2) show that both the a-tDCS stimulation and the s-tDCS groups significantly improved the feedforward activity of the lower extremity muscles in the single-leg landing skill after 12 intervention sessions (*P* < 0.05). However, the percentage of calculated changes indicated a more favorable effect of the anodal stimulation group. The results of between-group comparisons showed that the two study groups had a statistically significant difference in the single-leg landing skill in the feedforward activity of the Vastus Medialis (VM) (*P* = 0.01, η^2^_p_ = 0.18), Gluteus Medius (Gmed) (*P* = 0.03, η^2^_p_ = 0.18), Semitendinosus (ST) (*P* = 0.04, η^2^_p_ = 0.12), and Gastrocnemius (Gas) (*P* = 0.0001, η^2^_p_ = 0.36) muscles (Figs. 1,2).

### Feedforward knee muscles activity: double-leg landing task

The intra-group comparisons (Table 3) indicate that the amount of feedforward activity of the lower extremity muscles in both the a-tDCS stimulation and s-tDCS control groups improved significantly after 12 intervention sessions in the double-leg jump-landing skill (*P* < 0.05). The percentage of changes calculated in the two groups indicated a more favorable effect of the anodal stimulation group in improving the feedforward activity of the lower extremity muscles. Comparisons between groups in the mentioned task also demonstrated statistically significant differences in the feedforward activity of Gluteus Maximus (Gmax) (*P* = 0.014, η^2^ = 0.18), Gmed (*P* = 0.04, η^2^_p_ = 0.12), ST (*P* = 0.01, η^2^_p_ = 0.17), Biceps Femoris (BF) (*P* = 0.009, η^2^_p_ = 0.20), and Gas (*P* = 0.0001, η^2^_p_ = 0.41) muscles (Fig. 1,2).

### Feedforward knee muscles activity: lateral-hopping task

The amount of feedforward activity of the lower extremity muscles in the a-tDCS stimulation group and the s-tDCS control group improved significantly in the lateral-hopping task skill after 12 sessions (*P* < 0.05) (Table 4). The percentage of changes reflected the more favorable effect of the anodal stimulation group in improving the feedforward activity of the lower extremity muscles. However, there were statistically significant differences in the feedforward activity of, Gmed (*P* = 0.004, η^2^_p_ = 0.23), Gmax (*P* = 0.001, η^2^_p_ = 0.28), and Gas (*P* = 0.03, η^2^_p_ = 0.13) muscles between the two groups (Fig. 1,2).

**Table 4:** Within–between groups comparisons of feedforward activity of muscles for the the lateral hopping task (a-tDCS = s-tDCS, n = 17).

Muscles	Groups	Baseline	Four-week	CP	95% CI	Within group comparison	Between groups comparison	
		Mean ± SD	Mean ± SD			*P* value	*P* value η^2^_p_	
VL	a-tDCS	116.40 ± 41.87	155.80 ± 38.61	25.28	(− 53.84, − 24.97)	0.0001**	0.41	0.02
	s-tDCS	116.97 ± 39.11	134.32 ± 44.17	12.91	(− 26.10, − 8.60)	0.001**		
VM	a-tDCS	106.55 ± 49.19	150.34 ± 60.89	29.12	(− 58.86, − 28.72)	0.0001**	0.038	0.13
	s-tDCS	113.44 ± 48.90	124.83 ± 52.98	9.12	(− 18.51, − 4.27)	0.004**		
Gmax	a-tDCS	68.64 ± 36.68	99.55 ± 43.15	31.04	(− 42.84, − 18.95)	0.0001**	0.001**	0.28
	s-tDCS	64.84 ± 21.07	75.19 ± 18.53	13.76	(− 15.04, − 5.66)	0.0001**		
Gmed	a-tDCS	76.31 ± 37.44	110.26 ± 38.96	30.79	(− 49.69, − 18.20)	0.0001**	0.004**	0.23
	s-tDCS	63.10 ± 33.36	75.25 ± 33.12	16.14	(− 15.04, − 5.66)	0.0001**		
ST	a-tDCS	65.32 ± 34.74	91.81 ± 41.54	28.85	(− 37.95, − 15.01)	0.0001**	0.06	0.1
	s-tDCS	55.54 ± 24.46	64.40 ± 25.73	13.75	(− 13.03, − 4.67)	0.0001**		
BF	a-tDCS	56.82 ± 25.93	84.66 ± 40.79	32.88	(− 39.02, − 16.64)	0.0001**	0.14	0.06
	s-tDCS	61.50 ± 33.24	77.42 ± 39.72	20.56	(− 27.07, − 5.75)	0.004**		
Gas	a-tDCS	100.75 ± 37.04	136.80 ± 54.04	26.35	(− 52.54, − 19.55)	0.0001**	0.03**	0.13
	s-tDCS	80.05 ± 32.89	89.23 ± 34.39	10.28	(− 14.10, − 4.26)	0.001**		

All the muscles show the value of %MVIC; VL: Vastus lateralis; VM: Vastus Medialis; Gmax: Gluteus Maximus; Gmed: Gluteus Medius; ST: Semitendinosus; BF: Biceps Femoris; Gas: Gastrocnemius; CI: Confidence Interval; SD: Standard deviation; CP: Change percentage; ** = *P* < 0.01.

## Discussion

Decreased neuromuscular control of the trunk and lower limbs in women may increase the potential for DKV, and induce a higher rate of ACL injuries. Feedforward control of muscles is critical to provide dynamic knee joint stability in the face of joint and ligament injury mechanisms. The feedback control mechanism is not fast enough to be activated before the forces determining the injury have already exerted their deleterious action^20^, and DKV may cause a delay in the feedforward activation of the muscles protecting the knee. Several recent interventions have been introduced to prevent or correct DKV. However, no definite strategy has been reported to control and correct this functional defect^33^.

Our results showed that both real and sham transcranial stimulation groups can significantly reduce the DKV after interventions. However, no significant difference was observed between the two groups of real stimulation and sham in terms of the effectiveness. Neuromuscular training reduces the potential for ACL injury by improving biomechanical deficits commonly associated with injury^34,35^. Mayer et al. also showed that if NMT is implemented before puberty, it can reduce the risk of ACL injury in young female athletes^23^. Hopper et al. also confirmed that a 6-week NMT program can improve landing biomechanics associated with ACL injury in 11–13-year-old female netball athletes, all confirming the findings of the present study^22^. The possible reason that the addition of tDCS stimulation could not produce a statistically significant effect in improving DKV compared to the sham group can be attributed to the short duration of the intervention, the session of the stimulation, and the number of subjects. Therefore, it is recommended to conduct more studies with different protocols.

Both real and sham stimulation groups, after a 12-session period, significantly improved the level of feedforward activity of the muscles acting on the knee in single-leg landing and double-leg landing, and lateral hopping activities. Both groups received NMT, the intra-group improvement in feedforward activity of the studied muscles can be attributed to NMT exercises, which was in line with the findings of previous studies. Some review studies have reported that NMT can increase active stabilization of the knee and reduce the incidence of ACL injury in female athletes^34^. Neuromuscular training increases the sEMG activity of the medial hamstring muscles, and that maybe reduces the risk of dynamic valgus. This neuromuscular adaptation observed during side-cutting can potentially reduce the risk of non-contact ACL injury^36^. Yamamoto et al. (2015) in a similar study, investigated the effect of dynamic NMT on lower limb muscle activity on landing, and showed that NMT improves muscle activity before foot contact^37^. NMT for high-risk populations might reduce the risk of ACL injury and help female athletes to enjoy the benefits of sports participation without long-term disabilities^35^. Despite the significant intra-group effectiveness in both study groups, the percentage of improvements indicated that the anodal stimulation group was more effective in improving the feed-forward activities of the studied muscles. Few studies have been conducted regarding the effect of anodal tDCS on the feedforward activity of lower limb muscles. However, M1 stimulation improved the excitability of the corticospinal tract, and this caused an increase in muscle fibers recruitment. Bilateral M1 tDCS significantly improved muscle strength and explosive force of the non-dominant knee extensor and flexor, possibly from increased motor units’ recruitment^38^.

The two groups showed a statistically significant difference in the feedforward activity of the ST muscle in the single-leg landing skill after completing 12 intervention sessions. Since this muscle is located medially, increasing the feed-forward activity of this muscle can effectively reduce the DKV. The hamstrings can act as functional ACL synergists, so co-activation and/or pre-activation of the medial hamstring muscles (frontal plane antagonists to lateral quadriceps forces) may provide important stabilization and protection during landing and rapid cutting tasks^39,40^.

In addition, Gmed and VM muscles showed significantly better feedforward activity compared to the sham group, and these two muscles have been reported in different studies as muscles that control valgus and pressure on the ACL ligament. So that, Ueno et al. showed that the coupled hip adduction and lateral pelvic tilt were associated to the increased vertical and lateral ground reaction forces, propagating into higher knee abduction moments. These biomechanical features are associated with ACL injury and may be limited in a landing with increased activation of the Gmed^41^. Dan Wang et al., (2023), also mentioned the importance of hamstring and Gmed muscles activity in preventing knee abduction moment as an important ACL risk factor^42^. The lateral Gas muscle helps in creating the knee flexion moment and external rotation of the tibia, and this increase in muscle activity can have a positive effect on reducing the load on the ACL ligament. Therefore, using a-tDCS in combination with rehabilitation exercises and injury prevention can probably be effective in overloading the ACL ligament during unpredictable disturbances. In the double-leg landing task, the two groups significantly differed in the feedforward activity of the gastrocnemius, Gmed Gmax, ST, BF muscles after 12 intervention sessions. The Gmed muscle is one of the most effective muscles in controlling DKV and loading on the ACL ligament. The fewer fibers recruitment of Gmed muscle at the moment and before foot contact in landing and hopping skills, the ability of this muscle to control the femur in the frontal plane will decrease, and the probability of DKV will increase^43^. As mentioned in the single-leg landing, Dan Wang et al. reported the importance of hamstring muscles (BF, ST) and gluteus medius activity in preventing knee abduction moment as an important risk factor for ACL injury^42^. In this study, a-tDCS intervention significantly improved the feedforward activity of the Gmed and hamstring, therefore, it is possible adding a-tDCS to the exercises in professional athletes can improve the stability of the knee joint by reducing the knee abductor moment. In addition, the a-tDCS intervention improved the activity of the gastrocnemius muscle (a secondary knee flexor) after 12 sessions. As in previous studies, increasing knee flexion at foot contact with the ground during landing and hopping can reduce excessive loading on the ACL ligament^44^.

In the lateral hopping task, the feedforward activity of the Gmax, Gmed, and gastrocnemius muscles exhibited a significant difference between the real and sham stimulation groups in the post-test. The gluteus maximus and external rotators of the thigh can be effective in controlling the knee and preventing DKV by controlling the internal rotation of the femur, and the activation of the gluteus medius muscle allows to maintain the femur close to the midline^45^. Significant increase in the feedforward activity of the gastrocnemius muscle in the real stimulation group as knee flexor muscle may also exert a protective effect on knee stability. In addition to its secondary role as a knee flexor, the gastrocnemius may also contribute to the rotational moment of the knee, and is usually the first muscle group to be activated during rotational disturbances. Athletes with ACL injuries had exhibited a lower semitendinosus activity than healthy individuals^46^. The present study sheds novel light on the effects of tDCS in athletes with DKV and prevention of knee injuries, suggesting that tDCS in combination with NMT may enhance relevant feedforward electrical activity of lower limb muscles in athletes with DKV.

The present investigation has several limitations. For example, this study involved healthy young women athletes, which limits the generalization of our results. Future studies should involve subjects with knee injuries, different age groups, and men. The evaluator and trainer were the same in the current study and there was no blinding condition, therefore in future research, it is recommended to have different individuals as the evaluator and trainer to avoid any bias. To enhance the findings of the present study, incorporating motion analysis alongside sEMG could provide a more comprehensive understanding of the subject matter. The researchers in this study did not investigate the effect of tDCS alone, which was another study's limitations that can be examined in future studies.

## Materials and methods

### General experimental procedure

This randomized, sham controlled, single-blind clinical trial with parallel groups and a pre-test-post-test design was conducted in the Sports Rehabilitation and Corrective Exercises Laboratory of Razi University from August to October 2022.

### Participants

**Figure 3:**
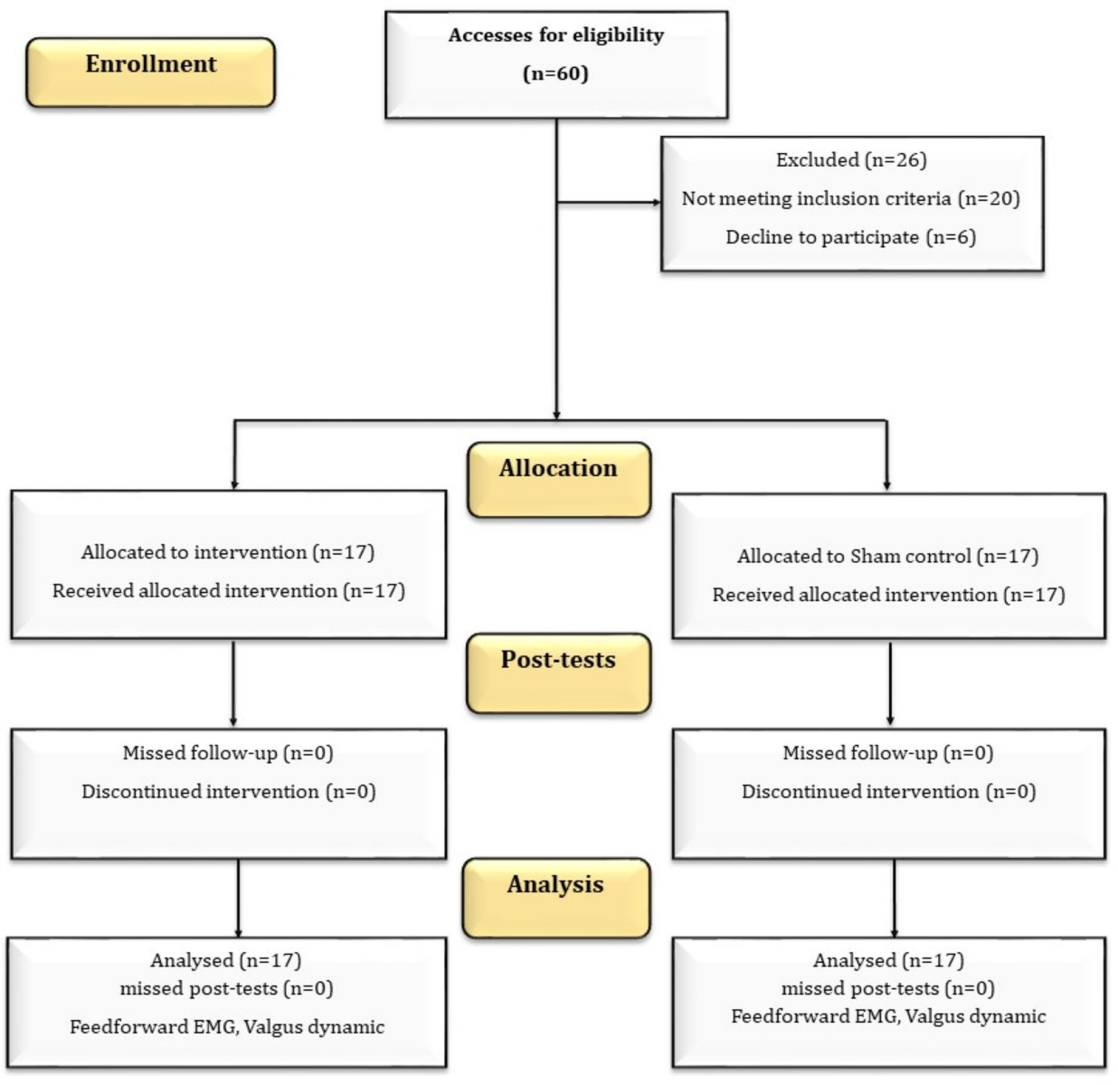
Study flow chart.

A total of 34 healthy elite martial athletes (Taekwondo) (mean age: 21.76; mean body mass index: 22.17; mean training history: 5.54 yrs.) with DKV during landing were invited to participate in the study. The inclusion criteria were age range of 15–30 years, healthy at the time of the study, at least three years of continuous and regular participation in combat training, and DKV of more than 12 degrees. Athletes with an acute injury in the last six months, a history of fracture or unresolved surgery in the lower limb, having a significant deformity in the lower limb, receiving other strengthening interventions, and neuromuscular diseases were excluded (Fig. 3). The sample size was calculated using G*Power software (Version 3.1.9.2, University of Dusseldorf, Germany) according to the following command: test family = F tests; Statistical test = ANCOVA: Fixed effects, main effects and interactions; α error probability = 0.05; power (1-b err prob) = 0.80; Effect size f = 0.5^47^, number of groups = 2, numerator df = 1, number of covariates = 1. Accordingly, 34 subjects were appropriate as the sample size of the present study.

### Randomization and blinding

Subjects were randomized by an individual out of the research using a random allocation software (version 1.0.0, Razi University of Medical Science, Iran). To do so, subjects were assigned numbers from 1 to 34, and each number was randomly assigned either to the experimental or to the control group by the software. Each number and group assigned to it were written on a sheet after randomization and placed in an opaque envelope by the very person who did the randomization. After the pre-test evaluations, each individual's envelope was opened, and the subject and evaluator were informed about the assigned group. The evaluator did not know about the subject's assigned group before the evaluations. The subjects did not know about the groups' existence because both groups' exercises were the same; one of the groups received real stimulation, and the control group received sham stimulation, therefore the research was single-blind.

### Procedure

After signing the written consent forms and completing general questionnaires, explanations about the testing procedures were given. Subjects were familiarized with the brain stimulation procedure, and to check any abnormal reactions to the brain stimulation, they received short doses of stimulation. The DKV was determined via a jump-landing test from a 30 cm box. The subject's jumping and landing were captured from the front, and the recorded video was evaluated with Kinoveia software version 0.9.5. Eligible subjects were randomly assigned to one of two groups: a-tDCS plus NMT (a-tDCS = 17) or NMT plus sham stimulation (s-tDCS = 17). The feed-forward activity of the lower extremity muscles (vastus medialis (VM), vastus lateralis (VL), Gluteus maximus (Gmax), Gluteus medius (Gmed), semitendinosus (ST), biceps femoris (BF), and gastrocnemius (Gas) in jump-landing tasks (single-leg and double-leg) and side hop was measured before and after warming up. Then, for four weeks, both groups received their respective interventions three times a week. Study outcomes were assessed immediately after completion of the interventions.

### Outcome measures

#### Dynamic knee *valgus*

**Figure 4:**
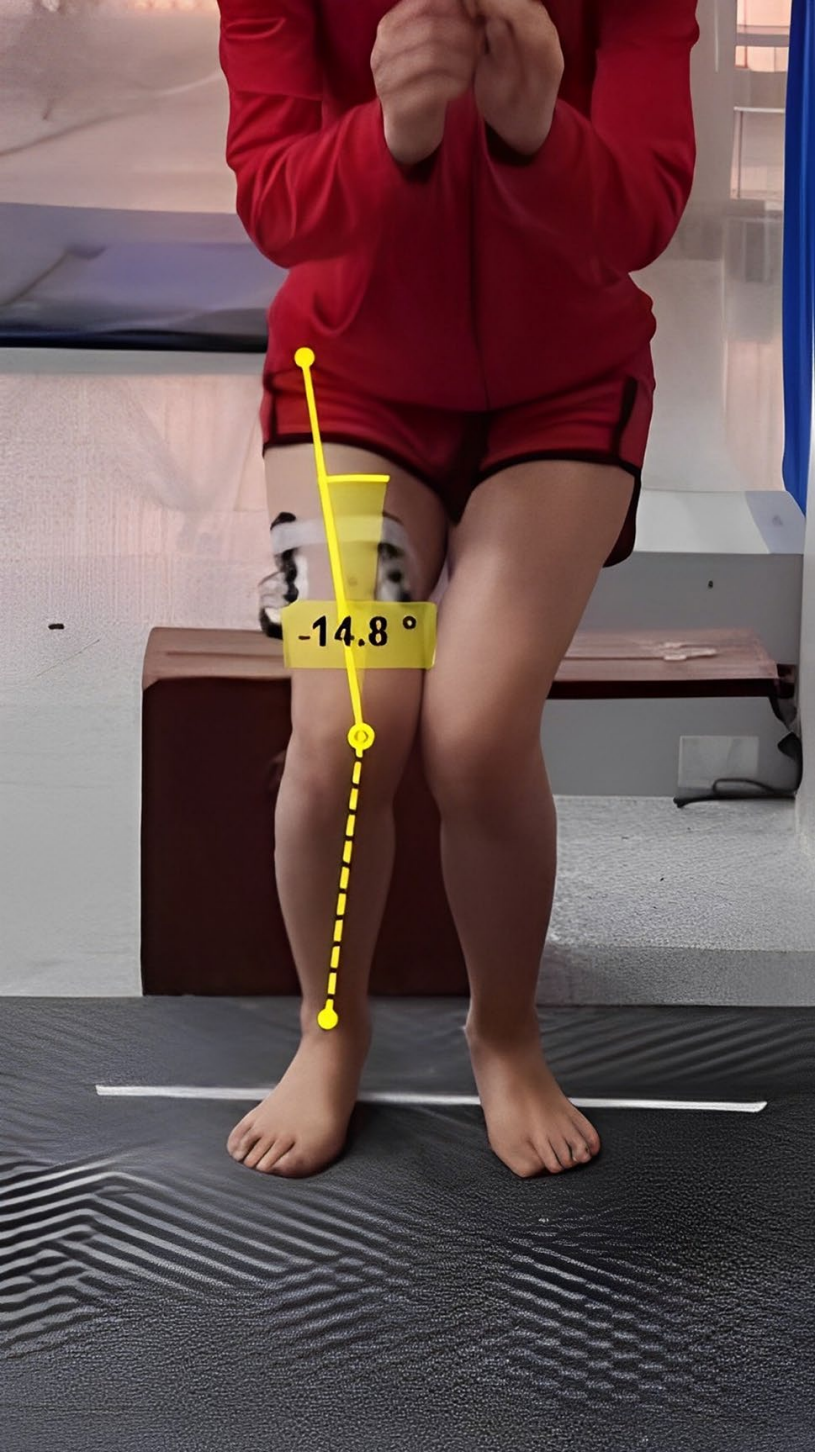
Valgus dynamic angle calculation with Kinovea software in double-leg landing.

The participant was required to begin on top of a 30-cm box with their feet shoulder width apart and their hands on their hips. The video frames recorded from the front view of jump-landing skills were examined. The landing moment frame, the frame in which the subject was at the lowest height, was selected, and the valgus angle of the knee was measured^48^. This angle was determined from the intersection of two lines drawn between the anterior superior iliac spine to the center of the patella and the line drawn from the center of the patella to the midpoint of the two ankles^49^. The valgus angle was measured from 180 degrees, and the knee valgus degree was determined. Kinovea version 9.0.5 software was used to measure the knee valgus angle. The jump-landing test was recorded for each subject three times with 30 s of rest between each landing, and the average angles of the three tests were used for analysis. If the dynamic valgus angle of the knee was greater than 12 degrees, the athlete was considered to have a DKV (Fig. 4)^49^.

#### Feedforward activity of muscles

**Figure 5:**
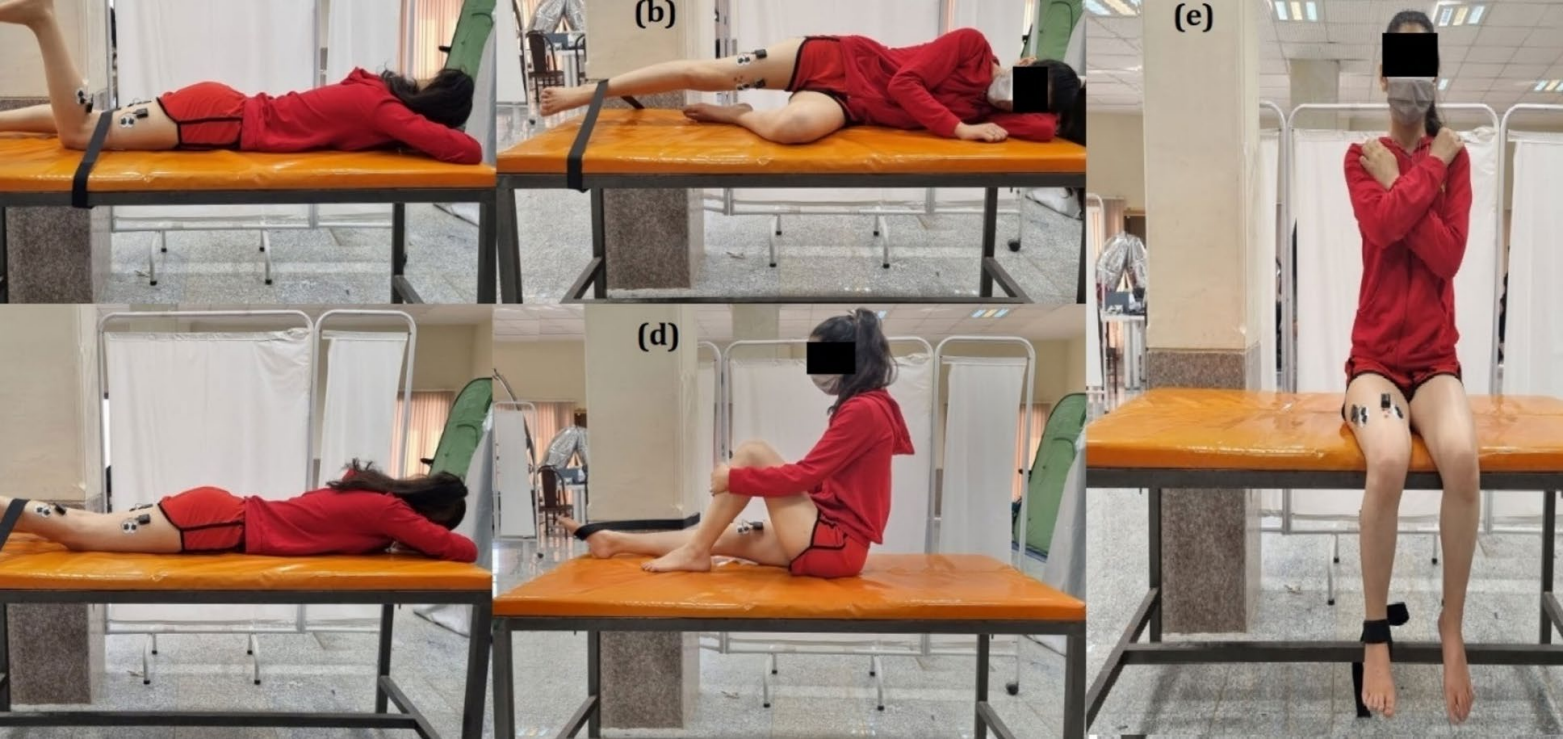
Maximum Voluntary Isometric Contraction (MVIC) tests for the six target muscles. (**a)** Gluteus maximus; (**b)** gluteus medius; (**c)** biceps femoris and semitendinosus; (**d)** lateral head of the gastrocnemius; (**e)** vastus medialis and vastus lateralis.

The activity of the subjects' leg muscles was recorded during landing and lateral hopping tasks using an eight-channel electromyography device (Noraxon, Ultium TM wireless sEMG system, Noraxon, Inc., Scotts-dale, AZ, USA). The skin was shaved after gentle rubbing with soft sandpaper and white alcohol to record raw muscle signals. Electrodes (Medico Electrodes, India) were placed on the skin with a center-to-center distance of 2 cm parallel to the muscle fibers based on SENIAM electrode placement recommendations^50,51^. The electrodes were positioned as follows: in the Gmax, they were placed at the 50% point of the line connecting the sacral and greater trochanter; in the Gmed, they were positioned in the middle of the line connecting the greater trochanter to the iliac crest; in the VM, they were positioned at the point where the anterior superior iliac spine (ASIS) met the anterior aspect of the medial knee ligament; and in the VL, they were positioned at the 2/3 point, In the BF, the electrodes were positioned halfway between the ischial tuberosity and the lateral epicondyle of the femur; in the ST, they were positioned halfway between the ischial tuberosity and the medial epicondyle of the femur; and in the gastrocnemius, they were positioned one point three lines from the head of the fibula to the heel. The Maximum Voluntary Isometric Contraction (MVIC) was used to normalize the electromyography data (Fig. 5). Resistance was applied to hip hyperextension to determine the MVIC of the Gmax^19,52^. MVIC of the Gmed was also recorded by performing an isometric contraction to abduct the hip^38^. In addition, resistance to hip extension was applied to the hamstring muscles with the athlete prone^52^. The person sat on the edge of the bed with the knee flexed to 90 degrees and made produced a maximum effort to exert the knee against a constant resistance^38,52^. MVIC of the gastrocnemius muscle was measured by ankle plantar flexion against a fixed resistance^53^.

The electromyography of the muscles was collected with a sampling rate of 1500 Hz per second. We investigated the feedforward activity of the lower extremity muscles and used 150 ms before the foot’s contact with the ground in landing and hopping activities as mark feedforward activities and for analysis. The Myo RESEARCH 3 software (Noraxon, Inc., Scottsdale, AZ, USA) was used to process and analyze the raw data. At first, a bandpass filter with a 20–450 Hz cutoff frequency was used. The data were rectified and processed using mean root square for 150 ms windows, normalized, and reported as a percentage of the maximum voluntary isometric contraction (%MVIC)^51^ (Eq. 1).

1 $$NEMG=\frac{{EMG}_{RMS}}{{MVIC}_{RMS}} \times 100$$

Subjects were asked to warm up for 10 min with walking, running, and jumping before performing jump-landing and lateral hopping tasks. Each participant was acquainted with the tests of single leg landing, double leg landing, and side hopping. In the jump-landing test, the subject stood on top of a 30 cm box with one leg in the air and hands on the waist and asked to jump with that foot down with the same leg as far as possible after touching the ground and at the end of this movement to land with the same leg^54^. In the lateral hopping test, the person stood on their dominant leg at a distance of 30 cm from the tape attached to the ground and then started the test from the side of that tape and performed a lateral jump. A 3D accelerometer (DTS 3D, Noraxon, USA) synchronized with the sEMG device was used to determine the time of foot contact with the ground.

### Study interventions

#### Neuromuscular exercises

**Figure 6:**
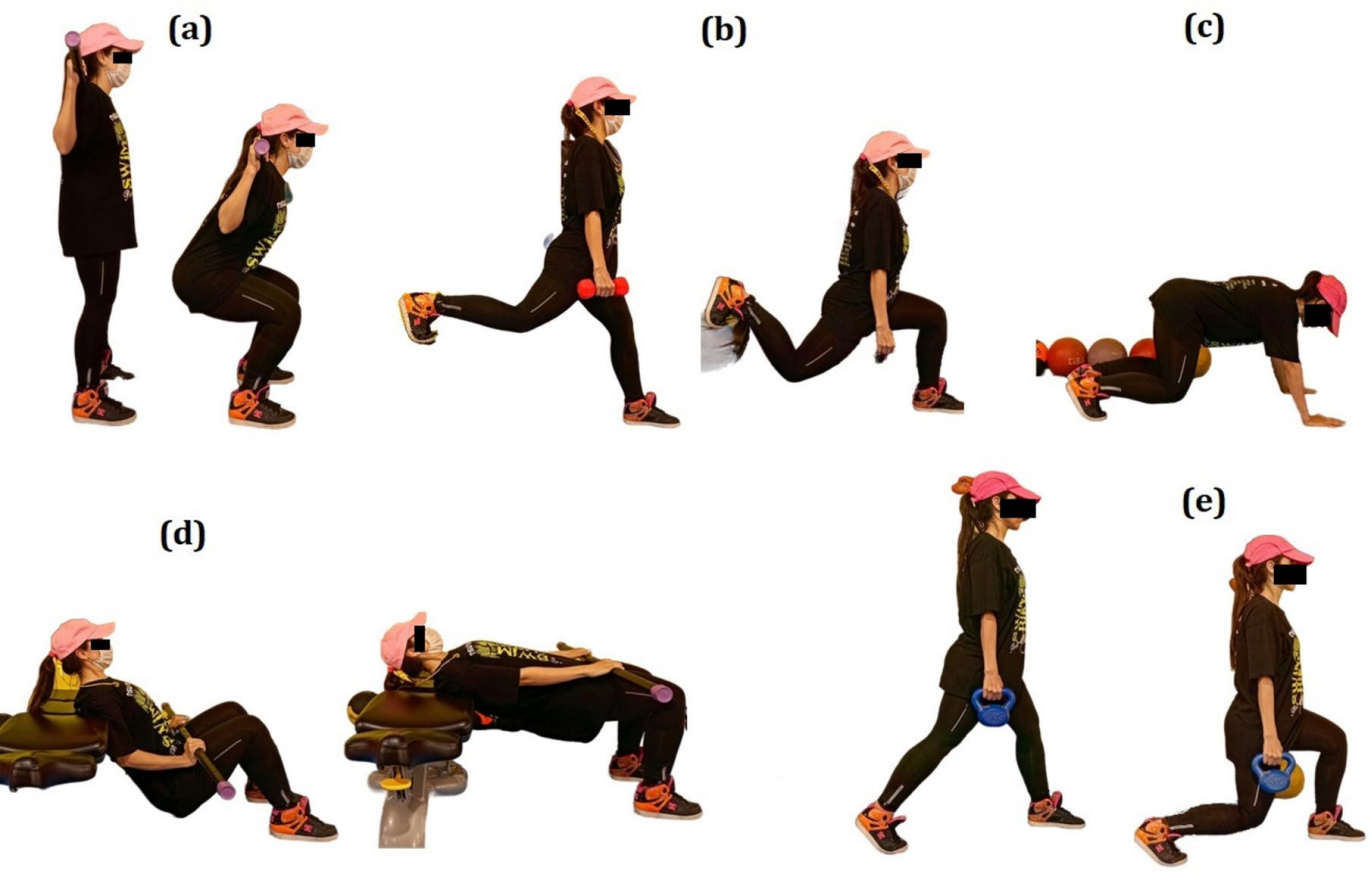
(**a)** Back squat; (**b)** DB split squats; (**c)** weighted bear crawl hold; (**d)**. hip thrusts; **e**. walking DB lunge.

The neuromuscular exercise protocol included 10-min of warm-up, 40-min of main body exercises, and 10-min cool-down. The training program was implemented from the program described by Dobbs et al., and consisted of four different exercise progressions that focused on resistance, plyometrics, dynamic stability, and core strengthening exercises^47^. We used free weight resistance exercises (for example, weight Lunges and Squats, Standing Band Rows, etc.), stretch–shortening cycles or jump training for plyometric exercises (such as Hurdle jumps and Split jumps), single-leg 90° hop-hold for dynamic balance stability^55^, and Pallof press and plank for example core was used strengthening exercise. The exercises were performed thrice a week for four weeks, and each session lasted 60 min. Each exercise was performed with 3–4 sets and 10–15 repetitions. The intensity of the exercises was gradually increased to apply progression and prevent adaptation. For this purpose, body mass, barbell, dumbbells, resistance bands, and weight plates were used. Subjects were trained for a week to familiarize with the protocol before starting the training. The exercises were performed by a certified sports injuries and corrective exercise coach with ten years of coaching experience (Fig. 6).

#### Transcranial direct current stimulation (tDCS)

A battery-driven stimulator (NeuroStim 2, Medina Tebgostar, Iran), international 10–20 system, and EEG Cap were used to precisely stimulate the target area in the brain (M1 region). Two carbon electrodes (5 × 4 cm; 20 cm^2^) covered with surface sponges soaked in saline solution (NaCl 140 mmol dissolved in Milli-Q water) were used as an anode and cathode. The electrodes were held in place using elastic bands. After coming to the laboratory, the subjects were asked to sit on the armchair in the prescribed position. Using a unique EEG cap, the electrodes were placed in the target areas that had previously been marked. The anode was placed over the right M1 (corresponding to FC2 according to the international 10–20 EEG system) and the cathode was placed over the left shoulder. The same montage was used for the sham condition in which the current was gradually increased for 30 s to create a state similar to anode stimulation and then, returned to the initial state so that no real stimulation was given. In each session, the subjects sat on an armchair without any verbal communication and received 20 min of the brain stimulation (anodal or sham based on their experimental groups) at 2 mA intensity^56^.

### Data analysis

SPSS software (Version 25.0, SPSS, Inc. Chicago, IL) was used for statistical data analysis. The level of significance was set at 0.05, and the confidence interval at 95%. At first, the Shapiro–Wilk and Leven’s tests were used to check the normal distribution of all data set and homogeneity of the variances, respectively. One-way analysis of covariance (AVCOVA) was used for between groups comparisons and the pre-test was set as a covariate variable. Partial eta squared (*ɳ*^*2*^_*p*_) was used as a measure of the effect size for the ANCOVAs and interpreted as small (0.01–0.059), medium (0.06 to 0.139), or large (≥ 0.14)^57^. Paired sample t-test was applied to analyze within-subject effect (pre to post). Equation 2 was used to calculate the percentage of change from the pre-test to the post-test in each variable:

2 $$Change\, percentage=\frac{posttest-pretest}{pretest} \times 100$$

## Conclusion

The results of the present study indicate that the addition of tDCS stimulation to the neuromuscular training did not lead to a significant change in DKV. However, it caused a significant improvement in the feedforward activity of the lower extremity muscles that are involved in knee movement during single & double-leg landing and lateral hopping tasks compared to the sham-control group. Consequently, therefore, it seems that more research is needed in this field by using other types of transcranial electrical stimulation (TES) in combination with exercises.

### Ethics approval

The Ethics Committee in Biological Research of Razi University and Iranian Clinical Trial Center approved the research process (Iran.REC.1401.003 & IRCT20220614055164N1). This investigation was conducted in agreement with the Declaration of Helsinki. All subjects signed a written consent form and were free to withdraw from the study at any time they chose to.

## Data Availability

The data that support the results of current study are available from the corresponding author on reasonable request.
